# Therapeutic Potential of *Rosmarinus officinalis* Extract on Endometriosis: Evidence from In Vitro Models

**DOI:** 10.3390/ijms27135654

**Published:** 2026-06-23

**Authors:** Sofía del Valle, Ignacio Edgardo Ruiz Arias, Gustavo Leirós, Mariela Bilotas, Nancy Adriana Espinoza-Sánchez, Burkhard Greve, Martin Götte, Analía Ricci, Gabriela Meresman

**Affiliations:** 1 Instituto de Biología y Medicina Experimental (IBYME), Consejo Nacional de Investigaciones Científicas y Técnicas (CONICET), Ciudad Autónoma de Buenos Aires C1428ADN, Argentina; sdelvalle782@gmail.com (S.d.V.); m.bilotas@ibyme.org.ar (M.B.); a.ricci@ibyme.org.ar (A.R.); 2 Instituto de Investigación en Medicina y Ciencias de la Salud, Universidad del Salvador, Ciudad Autónoma de Buenos Aires C1055AAG, Argentina; gleiros@usal.edu.ar; 3 Instituto de Ciencia y Tecnología César Milstein, CONICET—Fundación Pablo Cassará, Ciudad Autónoma de Buenos Aires C1440FFX, Argentina; iruiz@fundacioncassara.org.ar; 4 Department of Radiation Oncology, Münster University Hospital, 48149 Münster, Germany; nancyadriana.espinozasanchez@ukmuenster.de (N.A.E.-S.); burkhard.greve@ukmuenster.de (B.G.); 5 Department of Gynecology and Obstetrics, Münster University Hospital, 48149 Münster, Germany; mgotte@uni-muenster.de

**Keywords:** *Rosmarinus officinalis*, endometriosis, cell proliferation, cell migration, oxidative stress

## Abstract

Natural therapeutic alternatives are increasingly explored in endometriosis, a highly prevalent gynecological disorder with limited therapeutic options. *Rosmarinus officinalis* (rosemary) has attracted increasing scientific interest due to its biological activity. This study aimed to characterize a hydroethanolic rosemary extract (RE) and evaluate its effects on key cellular processes involved in endometriosis pathophysiology. Major phenolic compounds in RE were quantified by RP-HPLC, and antioxidant activity was assessed using DPPH, ABTS, and FRAP assays. After RE treatment, cell viability (WST-1), migration (wound healing assay), cell cycle distribution (DAPI staining), apoptosis (Annexin V/PI), p21 and cyclin A expression (Western blot), and intracellular ROS levels (DCFH-DA) were evaluated in endometrial stromal (t-HESC, St-T1b) and endometriotic epithelial (12-Z) cells. Phytochemical analysis revealed rosmarinic acid (RA) at 4.2%, while carnosic acid (CA) and carnosol (CS) together accounted for 23.7% of the extract. RE reduced cell viability and cell migration in 12-Z and t-HESC cells (*p* < 0.05). S-phase accumulation with a concomitant reduction in the G1 phase was observed across all evaluated cell lines (*p* < 0.05), along with increased p21 and cyclin A expression in stromal cells (*p* < 0.05). RE induced cell death in both 12-Z (*p* < 0.05) and St-T1b cells (*p* < 0.0001). In t-HESC cells, RE reduced both basal and H_2_O_2_-induced ROS levels (*p* < 0.01). These findings indicate that RE modulates key mechanisms involved in endometriosis pathophysiology, supporting its multi-target therapeutic potential as a nutraceutical approach for endometriosis management.

## 1. Introduction

Endometriosis is a highly prevalent chronic gynecological disorder, characterized by the ectopic growth of endometrial-like tissue outside the uterine cavity [1]. It is estimated to affect 10–15% of menstruating individuals, around 30% of those experiencing subfertility, and up to 80% of patients with chronic pelvic pain [2]. Conventional treatments, primarily involving hormonal therapy and surgical procedures, do not effectively address the underlying pathogenic processes. Furthermore, these approaches are associated with frequent side effects and uncertain impacts on fertility [3], highlighting the urgent need for safe and effective therapies that directly target the disease’s pathogenic mechanisms.

Although classified as a benign disease, endometriosis displays several hallmarks of neoplastic processes, including invasive behavior, resistance to apoptosis, and promotion of angiogenesis [4]. These similarities have prompted the exploration of oncological therapeutic frameworks for novel treatment approaches. In this context, phytotherapy, based on its long-standing use in traditional medicine, has gained recognition for its diverse bioactive compounds, cost-effectiveness, and relatively low risk of adverse effects compared with synthetic drugs [5].

Phytochemicals that regulate redox balance and cell proliferation may provide therapeutic benefits in endometriosis by counteracting its chronic inflammatory environment, characterized by local estrogen overproduction, oxidative stress, and immune dysregulation that promote lesion growth and neovascularization [6]. In agreement with this hypothesis, our group has previously evaluated several plant-derived compounds in experimental models of endometriosis, yielding promising outcomes [7,8,9,10].

*Rosmarinus officinalis*, commonly known as rosemary, is a widely used aromatic and medicinal plant cultivated in many regions worldwide [11]. It has received particular attention due to its potent antioxidant, anti-inflammatory, antinociceptive, and antiseptic activities [12,13,14,15]. Our previous in vitro and in vivo studies have shown that the isolated bioactive constituents of rosemary, carnosic acid (CA) and rosmarinic acid (RA), significantly inhibit the development of experimental endometriotic lesions [7]. Notably, these inhibitory effects are consistent with observations in a variety of tumor models, including colon, gastric, pancreatic, liver, lung, skin, and hematological malignancies, where rosemary polyphenols were found to suppress tumor growth and enhance the efficacy of conventional chemotherapeutics such as 5-fluorouracil [16].

Furthermore, a growing body of evidence indicates that crude plant extracts often exhibit greater antioxidant capacity than isolated compounds, an effect largely attributed to the combined action of multiple phytochemicals [17,18,19]. Building on our previous findings on the isolated rosemary constituents RA and CA, the present study evaluates for the first time a standardized whole hydroethanolic RE in endometriosis models to explore the integrated biological activity of its phytochemicals. When translating these findings to the treatment of complex human diseases, the application of phytotherapeutic approaches presents methodological challenges associated with the multicomponent nature of botanical extracts. Many plant-derived bioactive components exhibit low stability and limited in vivo bioavailability in their natural form, which can impact their clinical translation [20,21]. Additionally, achieving consistent analytical standardization remains a key consideration. Variations in active compound content between batches, often driven by plant genetics, harvest season, geographic origin, drying conditions, and extraction methods, can affect experimental reproducibility [22]. Despite these challenges, the biological effects of standardized RE have not yet been thoroughly investigated in endometriosis. Consequently, this study addresses an important gap in knowledge regarding its impact on endometriosis-relevant cellular mechanisms.

Based on these considerations, this study aimed to characterize a hydroethanolic extract of *Rosmarinus officinalis* and to comprehensively analyze its chemical composition, antioxidant activity, and its effects on key cellular processes involved in the pathophysiology of endometriosis using in vitro models. From a translational perspective, this approach may offer a distinct advantage by providing a more accessible and technologically scalable alternative for potential future development as a therapeutic or nutraceutical option.

## 2. Results

### 2.1. Phenolic Composition and Antioxidant Activity of RE

**Figure 1:**
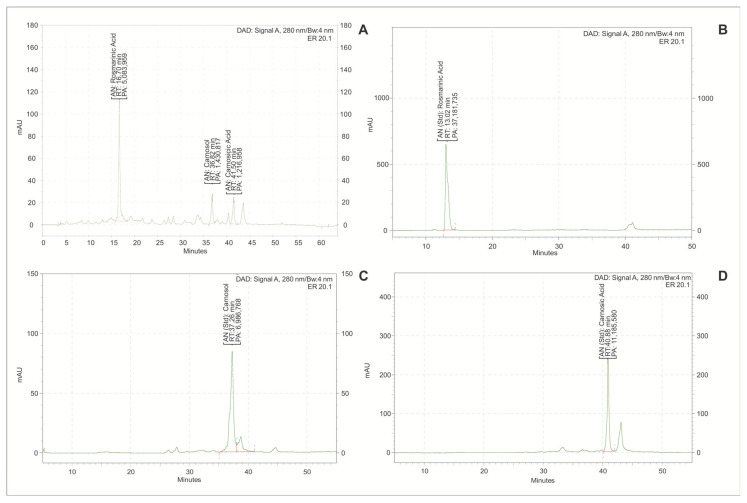
RP-HPLC analysis of RE phenolic compounds. Representative HPLC chromatograms of the RE (**A**) and reference standards: RA (**B**), CS (**C**), and CA (**D**). Signal intensity is expressed in milli-absorbance units (mAU) at 280 nm. For each peak, the analyte (AN), retention time (RT, min), and peak area (PA) are indicated.

Phenolic compounds were identified by comparing the retention times and UV absorption maxima of the extract peaks with those of reference standards, as described in the Section 4. The RP-HPLC chromatogram of the RE recorded at 280 nm revealed three major phenolic compounds, identified as RA, CS, and CA, with retention times of approximately 16.7, 36.8, and 42.5 min, respectively (Figure 1).

**Figure 2:**
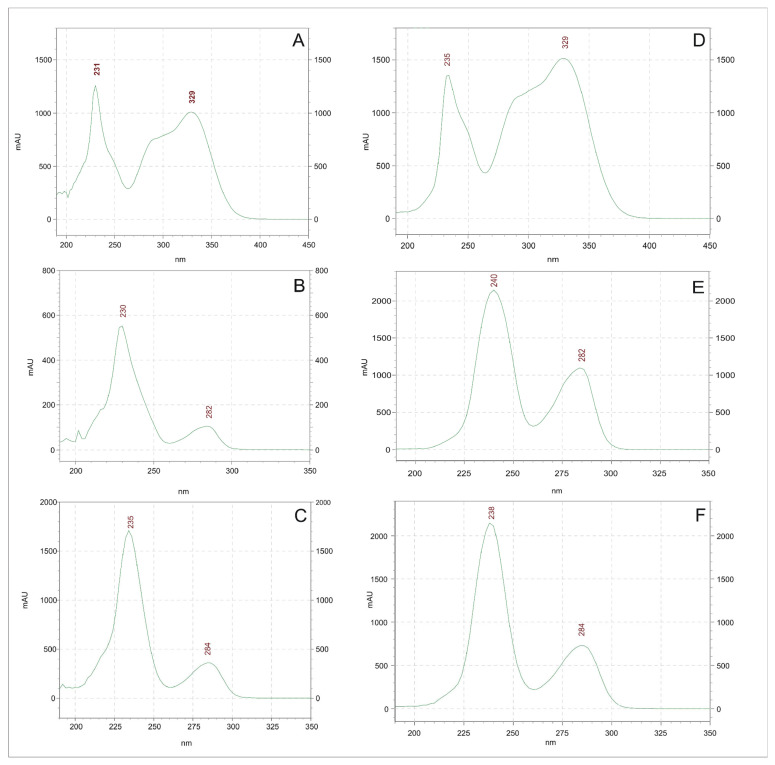
DAD UV spectra of phenolic compounds in RE. RA spectra are shown for RE (**A**) and the standard (**D**), CS for RE (**B**) and the standard (**E**), and CA for RE (**C**) and the standard (**F**). The wavelengths of maximum absorption are indicated at the respective peak maxima.

To further confirm compound identity, the UV spectra of each chromatographic peak were compared with those of the corresponding standards. The UV absorption profiles of the identified peaks closely matched those of RA, CS, and CA (Figure 2).

Quantification was carried out using calibration curves generated from serial dilutions of the corresponding reference standards (Supplementary Figure S1). The concentrations of each compound were calculated and expressed as a percentage of the extract dry weight. RA accounted for 4.24 ± 0.54%, CS for 9.63 ± 0.74%, and CA for 14.12 ± 3.50% of the extract. These results indicate that CA is the predominant polyphenol in RE, followed by CS and RA.

The extract exhibited antioxidant activities of 0.96 ± 0.02, 0.88 ± 0.02, and 1.07 ± 0.10 mmol TE/g dry RE in the ABTS, FRAP, and DPPH assays, respectively, indicating consistent antioxidant activity across the different methods employed.

### 2.2. RE Reduces the Viability of Endometrial Stromal and Endometriotic Epithelial Cells

**Figure 3:**
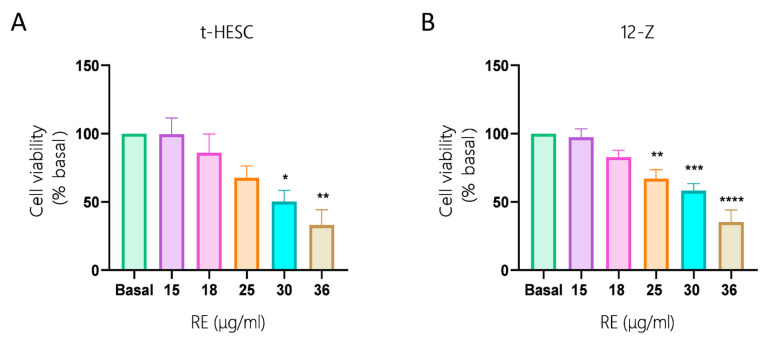
RE reduces cell viability. Cell viability of endometrial stromal (**A**) and endometriotic epithelial (**B**) cells after 24 h of treatment. Results are expressed as a percentage of basal values and represent the mean ± SEM. * *p* < 0.05, ** *p* < 0.01, *** *p* < 0.001, **** *p* < 0.0001 vs. basal.

As a first approach to evaluate the biological effects of RE, endometrial cell viability was examined. After 24 h of treatment, RE significantly reduced cell viability in the endometrial stromal t-HESC cell line at concentrations ≥ 30 µg/mL (Figure 3). A similar inhibitory effect was observed in endometriotic 12-Z epithelial cells at doses ≥ 25 µg/mL.

### 2.3. RE Promotes S-Phase Cell Cycle Accumulation in Endometrial Stromal and Endometriotic Epithelial Cells

To determine whether the RE-induced reduction in cell viability was associated with alterations in cell cycle regulation, cell cycle distribution was analyzed in endometrial stromal and endometriotic epithelial cells.

**Figure 4:**
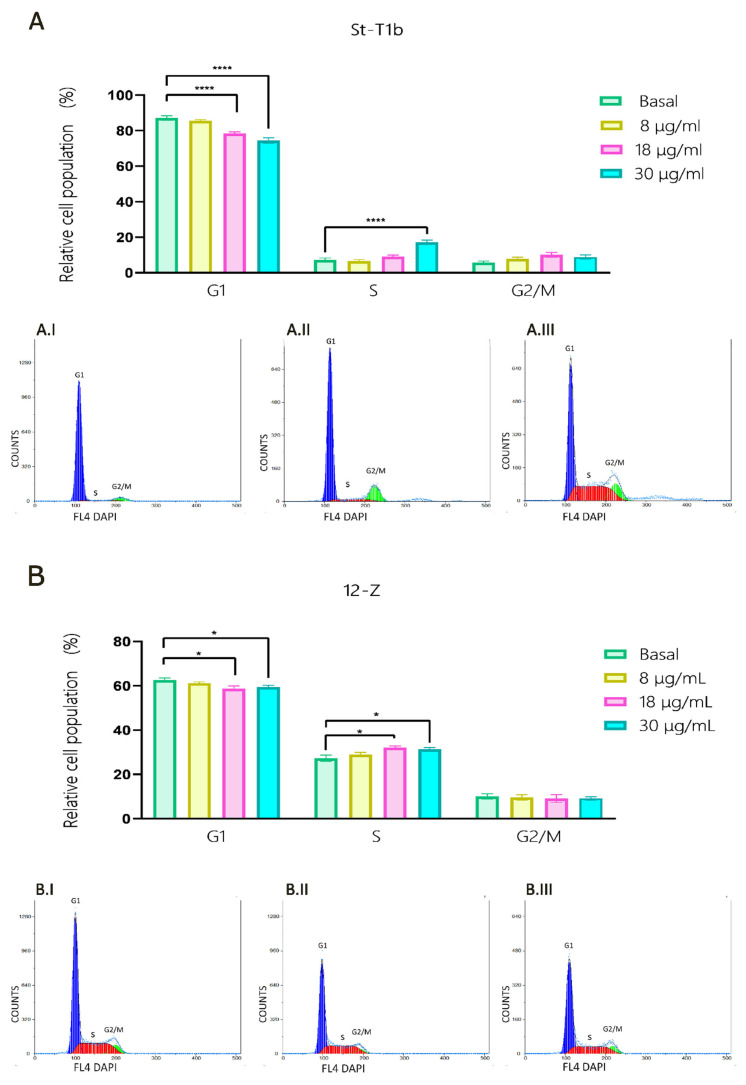
RE affects the G1 and S phases of the cell cycle. Quantification of cell cycle phase distribution by flow cytometry, expressed as relative cell population (%) in endometrial stromal (**A**) and endometriotic epithelial (**B**) cells after 24 h of RE treatment at the indicated concentrations. Representative histograms illustrating the distribution of cells in the G1 (blue), S (red) and G2/M (green) phases are shown for basal conditions (**A.I**,**B.I**) and for treatments with 18 µg/mL (**A.II**,**B.II**) and 30 µg/mL (**A.III**,**B.III**). * *p* < 0.05, **** *p* < 0.0001 vs. basal.

Treatment with RE at 18 and 30 µg/mL significantly reduced the percentage of cells in the G_1_ phase in both St-T1b and 12-Z cell lines (Figure 4). At 30 µg/mL, RE also altered cell cycle progression by increasing the proportion of stromal cells in the S phase. Similarly, in endometriotic epithelial cells, a significant increase in the S-phase population was observed following incubation with these doses (18 and 30 µg/mL).

Based on the observed RE-induced changes in cell cycle distribution, the expression of key cell cycle checkpoint regulators was subsequently evaluated. Specifically, protein levels of p21 and cyclin A2, which play critical roles in S-phase regulation, were assessed by Western blotting.

**Figure 5:**
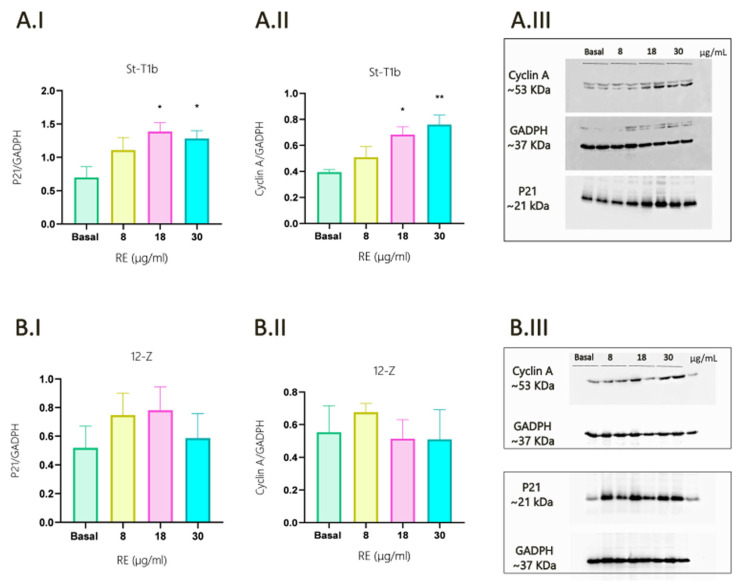
RE induces p21 and cyclin A2 expression in endometrial stromal cells. Western blot analysis of p21 and cyclin A2 expression in endometrial stromal (**A.I** and **A.II**, respectively) and endometriotic epithelial cells (**B.I** and **B.II**, respectively) treated with increasing concentrations of RE. Representative Western blot images of p21, cyclin A2, and GAPDH are shown for RE-treated endometrial stromal (**A.III**) and endometriotic epithelial cells (**B.III**). Results are expressed as mean ± SEM. * *p* < 0.05, ** *p* < 0.01 vs. basal.

Stromal cells displayed a significant increase in p21 and cyclin A2 expression at 18 and 30 µg/mL (Figure 5A), suggesting a possible association with the observed alterations in cell cycle distribution. In 12-Z cells, a similar trend toward increased expression of both proteins was observed; however, these differences did not reach statistical significance (Figure 5B).

### 2.4. RE Induces Cell Death at Higher Concentrations in Endometrial Stromal and Endometriotic Epithelial Cells

Given that alterations in cell cycle progression may be associated with the activation of cell death pathways, apoptosis was evaluated after 24 h of RE treatment in both cell lines using the Annexin V/FITC and PI assay.

**Figure 6:**
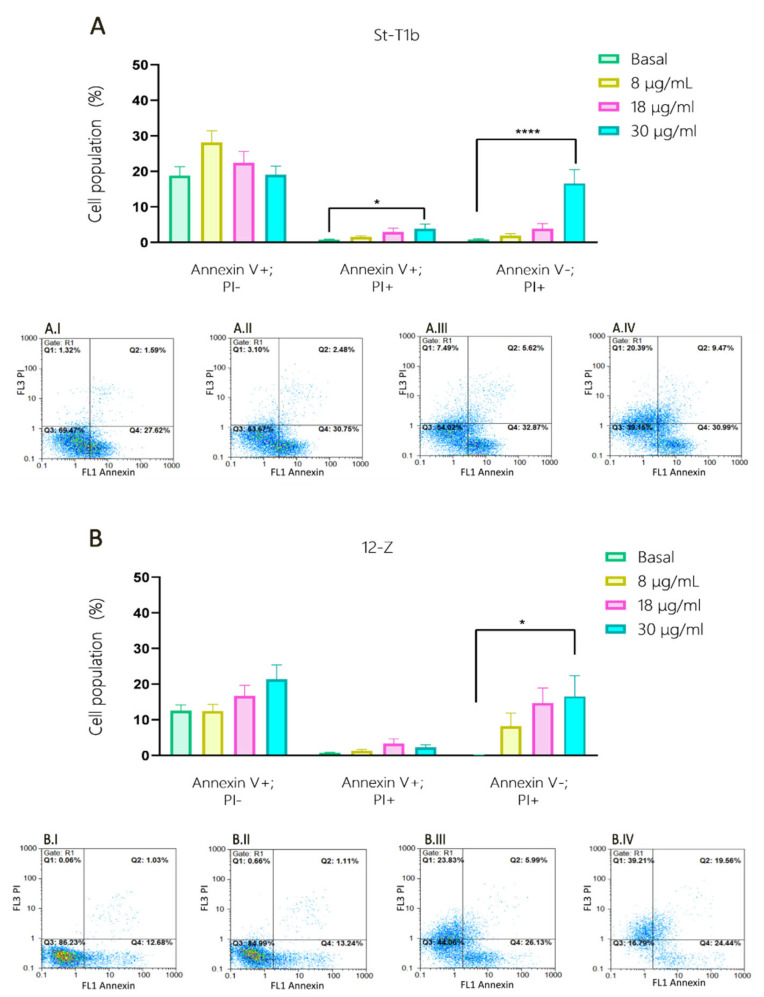
Apoptosis analysis by Annexin V/PI staining. Apoptosis in endometrial stromal (**A**) and endometriotic epithelial (**B**) cells after 24 h of RE treatment, evaluated by Annexin V/propidium iodide (PI) staining. Results are expressed as relative cell population (%) and represent the mean ± SEM. * *p* < 0.05, **** *p* < 0.0001 vs. basal. Representative flow cytometry dot plots showing the distribution of cell populations under basal conditions and following RE treatment are shown in (**A.I**,**B.I**) (basal), (**A.II**,**B.II**) (8 μg/mL), (**A.III**,**B.III**) (18 μg/mL) and (**A.IV**,**B.IV**) (30 μg/mL). Quadrants are defined as follows: Q1, Annexin V−/PI+ (necrotic cells); Q2, Annexin V+/PI+ (cells undergoing late-stage cell death); Q3, Annexin V−/PI− (viable cells); and Q4, Annexin V+/PI− (early apoptotic cells).

In endometrial stromal cells, a trend toward increased early apoptosis was observed following treatment with 8 µg/mL RE; however, this effect did not reach statistical significance. In contrast, the highest concentration tested (30 µg/mL) significantly increased the percentage of positive cells for both Annexin V and PI, representing a 19-fold increase compared with basal conditions, as well as cells positive for PI alone, compared with basal conditions, indicating late-stage cell death and primary necrosis, respectively (Figure 6A).

Similarly, in the endometriotic epithelial cell line, a non-significant trend toward increased early apoptosis was observed following treatment with 30 µg/mL RE. Notably, this concentration significantly increased the percentage of PI-positive cells, indicating enhanced cell death compared with basal conditions (Figure 6B).

### 2.5. RE Reduces Intracellular Oxidative Activity in Endometrial Stromal Cells

To assess the potential antioxidant activity of RE, intracellular oxidative activity was evaluated in the t-HESC and 12-Z cell lines after 24 h of treatment with increasing concentrations of RE using the fluorescent probe DCFH-DA.

**Figure 7:**
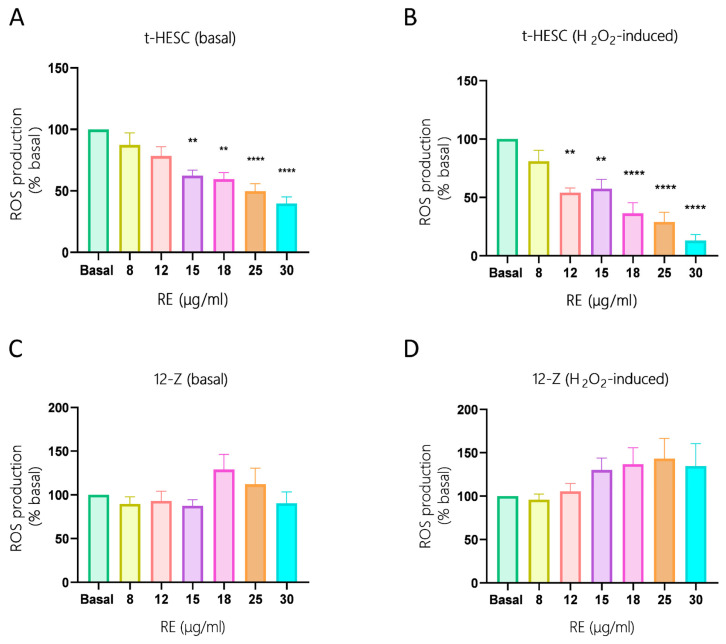
RE reduces ROS production in endometrial stromal cells. Intracellular ROS levels in endometrial stromal (**A**,**B**) and in endometriotic epithelial cells (**C**,**D**) under basal conditions (**A**,**C**) and following hydrogen peroxide stimulation (**B**,**D**) after 24 h of RE treatment, assessed using the fluorescent probe DCFH-DA. Results are expressed as a percentage of basal values and represent the mean ± SEM. ** *p* < 0.01, **** *p* < 0.0001 vs. basal.

RE treatment significantly reduced DCF fluorescence in t-HESC cells under both basal conditions (Figure 7A) and hydrogen peroxide-induced oxidative stress (Figure 7B). This effect was observed at concentrations ≥ 15 µg/mL in the absence of H_2_O_2_ and at concentrations ≥ 12 µg/mL in its presence. By contrast, RE treatment did not significantly modify DCF fluorescence in the endometriotic epithelial cell line (Figure 7C,D).

### 2.6. RE Inhibits the Migratory Capacity of Endometrial Stromal and Endometriotic Epithelial Cells

Cell migration was evaluated by a wound healing assay, and the percentage of wound closure was recorded at 0 and 20 h after treatment and analyzed as described above.

**Figure 8:**
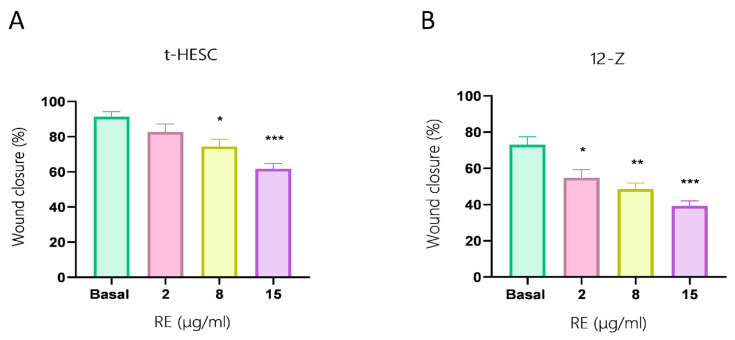
RE inhibits cell migration. Migration was evaluated as wound healing closure in endometrial stromal (**A**) and endometriotic epithelial (**B**) cells after 20 h of treatment. Data is expressed as mean ± SEM. * *p* < 0.05, ** *p* < 0.01, *** *p* < 0.001 vs. basal.

RE treatment significantly reduced the migratory capacity of both endometrial stromal (Figure 8A) and endometriotic epithelial (Figure 8B) cells. In t-HESC cells, a significant reduction in migration was observed starting at 8 µg/mL, whereas in 12-Z cells this effect was evident already at the lowest concentration tested (2 µg/mL).

Since wound healing assays may be influenced by proliferation, the scratch tests were carried out over a shorter period than the reported doubling time of both cell lines.

## 3. Discussion

Current pharmacological treatments for endometriosis are associated with important limitations, including undesirable side effects and a high rate of disease recurrence. Given the chronic and recurrent nature of the condition, there is a pressing need to develop therapeutic strategies with improved safety and efficacy profiles, minimal interference with reproductive function, and feasibility for long-term administration. In this context, natural compounds, whether isolated phytochemicals or multi-component herbal preparations, have attracted increasing scientific interest as potential therapeutic agents.

Several studies have shown that phytocompounds can modulate key pathophysiological processes implicated in endometriosis, including chronic inflammation, oxidative stress, and dysregulated cellular proliferation [6]. Rosemary, a widely consumed culinary herb with a long history of traditional use, has received growing scientific attention due to its broad pharmacological potential across a wide range of human conditions [23,24].

Based on this rationale, the present study aimed to characterize hydroethanolic RE and to evaluate its biological effects using in vitro models of endometriosis.

In this work, we obtained a stable hydroethanolic extract with a particularly enriched profile of bioactive compounds. Phytochemical analysis revealed a clear predominance of the two major diterpenes CA and CS, which together accounted for approximately 24% of the dry mass. These compounds, along with RA and other components present in the extract, have been individually associated with a variety of biological activities [16]. Previous studies from our group demonstrated beneficial effects of the rosemary constituents CA and RA in experimental models of endometriosis [7]. Building upon these findings, the present work moves beyond the evaluation of isolated constituents by investigating the biological activity of the whole RE, thereby providing a more comprehensive assessment of its therapeutic potential.

Although the individual contribution of each compound within the extract remains to be fully elucidated, the concurrent presence of both phenolic diterpenes and RA highlights the effectiveness of hydroethanolic extraction in preserving a broad spectrum of active constituents [25]. In line with previously published reports and with RE formulations approved by the European Food Safety Authority (EFSA), the concentrations observed in our study fall within the expected values for alcoholic preparations, in which the combined levels of the two primary antioxidant components typically range from 10% to 50% *w*/*w* [26,27,28]. For instance, a methanolic extract of *Rosmarinus officinalis* was reported to contain 30% CA and 16% CS, yielding a ratio of approximately 1.8:1, close to the 1.5:1 ratio observed in our extract and indicative of a diterpene profile associated with biological activity [14]. Supercritical fluid extracts have shown combined CA and CS levels from 11% to 21.4%, depending on extraction conditions [29,30], while a comprehensive review reported concentrations between 10 and 30% for CA and 1–3.8% for CS [31]. These results support the selection of this hydroethanolic extract for subsequent biological evaluation.

However, it is essential to note that direct comparisons of effective concentrations across studies are inherently limited, as the composition of rosemary extracts can vary substantially according to geographic origin, harvest time, and, most importantly, the extraction process employed. These methodological variations result in specific phytochemical profiles that hinder the linear comparison of doses between different botanical preparations, limiting comparative conclusions across the existing literature.

The active compounds of rosemary have well-documented antioxidant properties and act as regulators of key inflammatory pathways, including those controlling cell proliferation and migration, processes that are critically involved in the pathogenesis of endometriosis [11].

Accordingly, the RE evaluated in this study demonstrated marked antioxidant activity. Consistent with our findings, Shan et al. [32] and de Falco et al. [33] reported comparable antioxidant capacities for various common spice extracts, including *Rosmarinus officinalis*, with values of the same order of magnitude. Although antioxidant activity has consistently been reported for rosemary extracts, methodological differences among studies preclude direct quantitative comparisons [34].

To further explore the potential of the complete RE to modulate cellular processes relevant to endometriosis [7], we evaluated cell viability and found that it was reduced in both endometriotic and endometrial cells with increasing concentrations of the extract. Notably, Ferella et al. [7] reported antiproliferative effects in stromal cells of the isolated rosemary constituents CA and RA at concentrations of 10–25 µg/mL and 50–100 µg/mL, respectively. In contrast, in the present study, similar effects were observed with the whole extract at concentrations of 30 and 36 µg/mL, corresponding to 1.27–1.53 µg/mL RA and 4.24–5.08 µg/mL CA. Thus, comparable biological effects were achieved with substantially lower concentrations of the individual constituents than those previously reported for the isolated compounds. These findings suggest that the biological activity of RE may be driven by the combined action of its multiple phytochemical constituents, leading to greater potency than that observed for the isolated compounds.

Our observations align with previous reports on different rosemary formulations, mostly evaluated in cancer cell lines [23,35], although the potency of these extracts varies considerably depending on their composition, extraction method, and the cell type analyzed [11,31,36]. In breast cancer, a rosemary leaf extract was shown to dose-dependently inhibit the proliferation and clonogenic survival of MDA-MB-231 cells [37]. In prostate cancer, comparable antiproliferative effects were also observed in both androgen-independent and androgen-sensitive cells [38]. A supercritical RE rich in CA and CS decreased lung cancer cell proliferation [39], while in colon cancer, different extracts similarly suppressed proliferation in different cell lines [40,41]. More recently, a hydroethanolic extract rich in CS and RA reduced glioblastoma and rhabdomyosarcoma cell viability in a dose- and time-dependent manner [42].

The antiproliferative effects of rosemary extracts reported in various cancer models have been associated with cell-cycle modulation in a manner dependent on cell type and extract composition [40,43,44,45]. In our study, treatment with RE at 18 and 30 µg/mL increased the proportion of cells in S phase in both endometriotic epithelial and endometrial stromal cells, accompanied by a concomitant reduction in G1. The parallel upregulation of cyclin A and p21 in St-T1b cells suggests alterations in cell cycle regulation that may be associated with these specific checkpoints. While a similar non-significant trend was observed in 12-Z cells, the limited number of biological replicates may have contributed to the lack of statistical significance, restricting further mechanistic conclusions for this cell line. For St-T1b cells, our findings are consistent with those of Maya-Mendoza et al. [46], who demonstrated that p21 modulates replication fork speed and limits DNA synthesis during the S phase. Additional experiments would be required to definitively confirm a functional arrest; nevertheless, the observed S-phase accumulation was accompanied by molecular changes similar to those reported by Jiang et al. [47]. These authors observed an enrichment in the S phase, accompanied by increased cyclin A and decreased cyclin B expression, when evaluating the effects of rosmanol, a diterpene constituent of rosemary, suggesting that both whole extracts and individual bioactive compounds may converge on the regulation of S-phase progression. Similarly, Al Dhaheri et al. [48] demonstrated that CS alters cell-cycle progression in breast cancer cells through p21 upregulation.

Regarding apoptosis, treatment with RE showed a trend toward increased early apoptosis in 12-Z cells, although this effect did not reach statistical significance. In St-T1b cells, RE increased the proportion of cells undergoing late-stage cell death, with a significant effect observed at the highest concentrations tested. In both cell lines, the highest concentration of RE increased the proportion of necrotic cells. This finding reflects the variable apoptotic responses often observed with natural extracts in a concentration-dependent manner [31,40,49]. Similar cytotoxic effects have been reported previously for RE and other plant-derived compounds [40,49], suggesting that a threshold concentration may be required to trigger advanced apoptotic mechanisms. While previous studies have reported induction of early apoptosis in certain tumor cell lines exposed to RE or its principal constituents [50], our findings align with those of Pérez-Sánchez et al. [40] who observed no increase in early apoptosis but a marked rise in Annexin V/7-AAD double-positive cells in colon cancer cell lines, suggesting that RE promotes late apoptotic or necrotic death in a dose-dependent manner.

The antiproliferative activity of many phytochemicals has been linked to redox regulation [51], and several rosemary-derived compounds have been shown to modulate oxidative stress [52]. Considering these observations and other published evidence [53], it is plausible that cell death in our model may involve ROS-mediated mechanisms.

In our study, RE significantly reduced DCF fluorescence in endometrial stromal cells, whereas no significant modulation was observed in endometriotic epithelial cells, suggesting that the effects of RE on intracellular oxidative activity may be cell type-specific rather than representing a generalized response. Similar effects were reported in endometrial stromal cells treated with RA, where a role for ROS modulation in the regulation of cell proliferation was suggested [7]. In that previous work, isolated RA reduced intracellular ROS levels at concentrations of 50–100 µg/mL [7]. By comparison, the present RE achieved similar effects on intracellular oxidative activity at concentrations of 12–30 µg/mL, corresponding to only 0.51–1.27 µg/mL RA. These findings are consistent with the enhanced biological activity observed for the complete extract compared with its isolated constituents. Likewise, RE exhibits antioxidant activity in melanoma cells [43], and its main constituents, CA and CS, have been shown to reduce ROS levels in diverse pathological contexts [54,55]. Given that endometriosis is characterized by chronic inflammation and elevated ROS, these observations do not exclude a potential contribution of the antioxidant properties of RE to anti-inflammatory effects in a context-dependent manner. Importantly, as DCFH-DA is a nonselective indicator of overall oxidative status [56], these findings reflect a reduction in general intracellular oxidative activity rather than a definitive attenuation of specific pathways.

To further explore key mechanisms underlying endometriosis pathogenesis, we focused on cell migration, an essential process driving the establishment and expansion of ectopic lesions [57]. The significant reduction observed in the wound healing assay indicates that RE directly impairs cell migratory capacity. These results are consistent with accumulating evidence that natural compounds can modulate migration and invasion during endometriosis progression [8,57,58]. Similarly, multiple studies have demonstrated that RE suppresses the migratory activity of various cancer cell lines [38,40], supporting its potential role in this process.

It is noteworthy that the significant effects of RE on cell behavior were dose-dependent. Cell migration was significantly impaired at low concentrations (2–8 µg/mL), whereas the regulation of redox balance and the impact on cell cycle became apparent at intermediate concentrations (12–18 µg/mL). In contrast, the reduction in cell viability and the induction of cell death required concentrations of 25 µg/mL or higher. Considering that cell migration was inhibited at concentrations that did not substantially affect cell viability, the anti-migratory activity of RE does not appear to be merely a consequence of generalized cytotoxicity but a specific biological modulation of cell motility. In addition, alterations in cell cycle progression are likely to represent an upstream regulatory event contributing to the subsequent reduction in cell proliferation and viability, rather than a secondary consequence of cell death. Thus, while the inhibition of migration and the alterations in cell cycle progression observed at lower concentrations are consistent with specific regulatory effects, the reductions in viability and the induction of apoptosis observed at higher concentrations likely reflect a broader cytotoxic response.

Although this study provides promising evidence of the biological effects of RE in attenuating endometriosis-associated pathophysiological processes, further investigation is required to confirm its translational relevance. These effects were evaluated exclusively in vitro using monoculture models derived from immortalized cell lines. While these systems are highly informative for mechanistic exploration, they do not fully reproduce the cellular heterogeneity, extracellular matrix interactions, and complex hormonal, immune, and inflammatory microenvironment associated with endometriotic lesions. In addition, although representative markers of cell cycle regulation and apoptosis were evaluated, the precise upstream signaling pathways and molecular mediators responsible for the observed effects remain to be elucidated. To address these limitations, future in vivo validation incorporating pharmacokinetic, bioavailability, and safety assessments will be required to determine whether the evaluated concentrations in vitro are physiologically achievable.

From a broader translational perspective, compared with the complex and expensive isolation of pure chemical compounds, utilizing a standardized RE may offer a potentially enhanced activity through the combined action of its constituents while remaining a cost-effective and accessible alternative. This balanced profile makes RE an attractive candidate for further preclinical evaluation in endometriosis.

## 4. Materials and Methods

### 4.1. Materials

All reagents, unless otherwise stated, were purchased from Sigma-Aldrich Chemie GmbH (Taufkirchen, Germany).

### 4.2. Plant Material and Extract Preparation

The RE was kindly provided by Eurolab (Buenos Aires, Argentina). It was obtained from dried leaves of *Rosmarinus officinalis* cultivated in San Luis, Argentina, by hydroethanolic extraction followed by lyophilization, yielding a dry extract.

For experimental use, the extract was dissolved in 99.99% ethanol at a concentration of 333.33 mg/mL (based on total dry weight), resulting in a final dilution of 12%, and was stored in light-protected microtubes throughout the assays. Vehicle controls containing the same final ethanol concentration as that present in the highest RE dose were included in all experiments. No effects of the vehicle alone on cell morphology or viability were observed.

### 4.3. HPLC Analysis

The quantification of the major active phenolic compounds in a hydroethanolic extract of *Rosmarinus officinalis* was performed using reversed-phase high-performance liquid chromatography (RP-HPLC). Analyses were carried out on an Agilent 1200 HPLC system (Agilent Technologies, Santa Clara, CA, USA) equipped with an autosampler, quaternary pump, and diode array detector (DAD), using a Jupiter C18 column (Phenomenex, Torrance, CA, USA; 5 μm, 300 Å, 250 × 4.6 mm).

A continuous gradient elution of 5–80% acetonitrile in water containing 2.5% acetic acid was applied at a flow rate of 0.8 mL/min. The injection volume was 10 μL for both standards and samples. Chromatograms were acquired at 280 and 330 nm. Quantification was performed by calculating peak areas after HPLC separation and comparing sample chromatograms with calibration curves generated from serial dilutions of the corresponding reference standards.

The RE was dissolved in ethanol at a concentration of 20 mg/mL and filtered through a 0.45 μm membrane filter prior to HPLC analysis. Standard solutions were prepared gravimetrically and dissolved in ethanol at concentrations ranging from 0.05 to 2 mg/mL. The analyzed phenolic compounds were identified as RA, CA and CS using authentic reference standards (purity ≥ 96%) purchased from Enzo Life Sciences (Farmingdale, NY, USA). Calibration curves are provided in the Supplementary Figure S1.

Repeated phytochemical analyses performed over a two-year period showed comparable chromatographic profiles, indicating a consistent chemical composition throughout the experimental period.

### 4.4. In Vitro Antioxidant Capacity Assessment

The in vitro antioxidant capacity of the RE was assessed using three established methods: ferric reducing ability of plasma (FRAP) assay, 2,2-azino-bis-3-ethylbenzothiazoline-6-sulfonic acid (ABTS) radical-scavenging assay, and 2,2-diphenyl-1-picrylhydrazyl (DPPH) radical-scavenging assay [59].

All analyses were conducted in triplicate using Trolox as the standard antioxidant, and results were expressed in millimoles of Trolox equivalents (mmol TE) per gram of extract.

#### 4.4.1. FRAP Assay

The FRAP working reagent was prepared by mixing acetate buffer (pH 3.6), 10 mM tetrazolium red in 40 mM HCl, and 20 mM FeCl_3_·6H_2_O in a 10:1:1 ratio. To perform the assay, 3 mL of the working reagent was combined with 100 µL of a properly diluted sample. After a 30 min incubation in the dark, the absorbance was measured at 593 nm [60].

#### 4.4.2. ABTS Assay

The ABTS•^+^ radical cation was generated by reacting 7 mM aqueous ABTS with potassium persulfate (final concentration, 2.45 mM) and incubating the mixture in the dark at room temperature for 16 h. After this period, the ABTS•^+^ solution was diluted with ethanol to obtain an absorbance of 0.70 ± 0.02 at 734 nm.

To assess ABTS radical-scavenging activity, 3 mL of the ABTS•^+^ solution was transferred to a cuvette, and the initial absorbance (A_0_) was recorded at 734 nm. Subsequently, 100 µL of an appropriately diluted sample was added and mixed for 10 s, and the absorbance (A_1_) was measured exactly 3 min later at the same wavelength. Antioxidant activity was calculated as the change in absorbance (ΔAbs = A_1_ − A_0_), as described by Re et al. [61].

#### 4.4.3. DPPH Radical-Scavenging Assay

DPPH is a stable free radical that exhibits a strong purple color in methanol, which diminishes upon reaction with antioxidant compounds, indicating radical-scavenging activity. The assay was performed by incubating samples with DPPH solution (0.2 mM) in the dark for 15 min, after which the absorbance was measured at 517 nm [62].

### 4.5. Cell Lines and Culture Conditions

The immortalized endometriotic epithelial-like cell line 12-Z, derived from a peritoneal lesion and extensively characterized by Zeitvogel et al. [63], was maintained in a 75:25 (*v*/*v*) mixture of DMEM high-glucose and Ham F-12 supplemented with 10% fetal bovine serum (FBS) (Biochrom GmbH, Berlin, Germany) and 1% L-glutamine.

The human endometrial stromal cell line St-T1b, established from normal proliferative endometrial tissue and validated for its functional similarity to primary stromal cells [64], was cultured in DMEM high glucose, supplemented with 10% FBS, 5 μg/mL insulin, 2 mM L-glutamine, 1 mM non-essential amino acids, and 1% sodium pyruvate (100 mM).

The t-HESC (CRL-4003) cell line was obtained from the American Type Culture Collection (ATCC, Manassas, VA, USA). t-HESC, a telomerase-immortalized human endometrial stromal cell line that maintains the karyotypic and morphological features of primary stromal cells [65], was cultured in DMEM/F12 medium supplemented with 10% FBS and 1% L-glutamine.

The 12-Z, St-T1b, and T-HESC cell lines are widely used and extensively characterized models that retain key phenotypic and functional features relevant to endometriosis research, making them suitable tools for investigating disease-related mechanisms in vitro [66].

### 4.6. Treatment Conditions and Concentration Selection

The 24 h treatment period used in most experiments was selected based on previous studies from our group and others demonstrating that this timeframe, within controlled dosage ranges, is suitable for detecting biological and molecular responses to botanical compounds in endometriosis cell models [7,9,67].

The concentration range for the cell viability assay (15–36 µg/mL) was established based on preliminary in vitro experiments utilizing commercially available RA and CA [7] to evaluate a broad spectrum of concentrations. Based on these initial viability results, which revealed the onset of cytotoxicity at the upper limits of the range (30 µg/mL for stromal cells and 25 µg/mL for epithelial cells), subsequent sublethal dose ranges were strategically selected for further assays. Specifically, a range of 8–30 µg/mL was utilized for cell cycle, apoptosis, and intracellular ROS determinations to also study the biological effects at sub-lethal concentrations. Furthermore, a lower range of 2–15 µg/mL was strictly applied to the wound healing assay to ensure that the findings reflected genuine cell migration rather than alterations in cell viability or proliferation.

### 4.7. Cell Viability Assay

The viability of endometrial cells was analyzed using a colorimetric Water-Soluble Tetrazolium Salt-1 (WST-1, Roche Diagnostics GmbH, Mannheim, Germany). This assay is based on the ability of viable cells to cleave tetrazolium salts by mitochondrial dehydrogenases.

t-HESC (1 × 10^4^ cells/well) and 12-Z (1 × 10^4^ cells/well) cells were seeded in 96-well plates and maintained in complete medium for 24 h or until a 70–80% confluence was achieved. Then, cells were treated with RE at different doses (15, 18, 25, 30, and 36 µg/mL) under starvation conditions (1% FBS) for 24 h. Basal conditions were defined by the ethanol concentration corresponding to the highest RE concentration tested (0.18%). Following incubation, the treatment was removed, and WST-1 was added to each well and incubated for 2 h to allow color development. Absorbance was measured at 450 nm with background correction at 620 nm, using a microplate reader.

Cell viability was expressed as a percentage relative to the basal control condition. All assays were conducted in quadruplicate (t-HESC, *n* = 6; 12-Z, *n* = 5).

### 4.8. Cell Cycle Analysis

For cell cycle analysis, 3 × 10^5^ St-T1b or 12-Z cells per well were seeded in 6-well culture plates in medium supplemented with 10% FBS. After 24 h, cultures were washed and incubated for an additional 24 h with RE (8, 18, and 30 μg/mL) in medium supplemented with 1% FBS. All treatment groups were compared with the basal condition, treated with vehicle only.

Cell cycle progression was subsequently assessed by flow cytometry. Cellular DNA was stained with 4′,6-diamidino-2-phenylindole (DAPI; CyStain, Sysmex/Partec, Görlitz, Germany), and fluorescence intensity was measured according to the method described by Kahl et al. [68]. Cell cycle distribution was analyzed using FloMax software (version. 2.8. Quantum Analysis, Münster, Germany) and expressed as percentages of the total cell population (%). All assays were performed in triplicate (St-T1b, *n* = 5; 12-Z, *n* = 5).

### 4.9. Western Blotting

St-T1b and 12-Z cells were subjected to Western blot analysis following the procedure previously described [8,69].

After 24 h of treatment with RE (8, 18, or 30 μg/mL) or vehicle, cells were harvested and lysed in 100 μL of 2× Laemmli buffer supplemented with 100 mM dithiothreitol (DTT). Cell lysates were sonicated for 3–5 cycles of 5 s at 60% amplitude using an Ultrasonic Processor UP100H (Hielscher GmbH, Hamm, Germany) to obtain total protein extracts. Protein concentrations were determined using the Bradford assay [70].

Equal amounts of protein (15–30 μg per sample) were separated on 12% SDS–PAGE at 120–150 V and electrotransferred onto Protran Premium nitrocellulose membranes (GE Healthcare Life Sciences, Solingen, Germany) for 1 h at a constant voltage of 16 V.

Membranes were blocked and incubated overnight at 4 °C with the following primary antibodies: anti-p21 (rabbit, N2947; Cell Signaling Technology, Danvers, MA, USA; 1:5000), anti-cyclin A2 (rabbit, ab181591; Abcam, Cambridge, UK; 1:2000), and anti-GAPDH (mouse, sc-47724; Santa Cruz Biotechnology, Dallas, TX, USA; 1:10,000), used as a loading control. After washing five times with Tris-buffered saline, membranes were incubated with horseradish peroxidase-conjugated secondary antibodies: goat anti-rabbit IgG (1:5000) or goat anti-mouse IgG (1:10,000).

Chemiluminescent signals were detected using SuperSignal^®^ West Pico Chemiluminescent Substrate (Thermo Scientific, Rockford, IL, USA) and captured with a FUSION SL imaging system (Vilber Lourmat, Marne-la-Vallée Cedex, France). Band intensities were quantified using NIH ImageJ software (version 1.54g, National Institutes of Health, Bethesda, MD, USA), and densitometric values were normalized to GAPDH. Experiments were performed in triplicate for each protein and condition, and results were expressed as arbitrary units (St-T1b, *n* = 4; 12-Z, *n* = 3).

### 4.10. Apoptosis Assessment

Apoptotic cell death was assessed after a 24 h treatment of St-T1b or 12-Z cells with RE (8, 18, or 30 μg/mL) or vehicle (basal). Apoptosis was detected using the FITC Annexin V Apoptosis Detection Kit (BD Pharmingen, Heidelberg, Germany), which identifies phosphatidylserine externalization, an early hallmark of apoptosis. Cells were counterstained with propidium iodide (PI) to discriminate late apoptotic or dead cells.

Fluorescence emissions were recorded at 527 nm (FITC) and 675 nm (PI) using a CyFlow^®^ Space flow cytometer (Partec, Münster, Germany). Staining and acquisition procedures were performed according to previously described protocols [8]. Each treatment condition was analyzed in at least four independent experiments, and results were expressed as relative cell population (%) and compared to the basal condition.

The Annexin V/PI assay is typically evaluated using a four-quadrant model to categorize cells as viable (Annexin V−/PI−), early apoptotic (Annexin V+/PI−), late apoptotic (Annexin V+/PI+), or necrotic (Annexin V−/PI+). However, since the Annexin V+/PI+ fraction can include alternative forms of regulated cell death, and more specific methods to confirm apoptosis, such as caspase-3 activity or TUNEL assays, were not evaluated here, these cells were conservatively classified as undergoing late-stage cell death [71].

### 4.11. Wound Healing (Scratch) Assay

The wound healing assay was performed as previously described [8]. t-HESC and 12-Z cells (5 × 10^4^ cells/well) were seeded in 24-well culture plates and grown in complete medium until they reached 100% confluence. A uniform scratch was generated using a 100 μL pipette tip, and detached cells were removed by washing with phosphate-buffered saline.

Immediately after wounding, RE at concentrations of 2, 8, or 15 μg/mL, or vehicle, was added in culture medium supplemented with 1% FBS for t-HESC cells and 2% FBS for 12-Z cells. Scratch closure was monitored by phase-contrast light microscopy, and images were captured at 0 h (immediately after scratching) and 20 h post-wounding. The assayed migration time (20 h) was shorter than reported doubling times of both cell lines (t-HESC: 32–42 h; 12-Z: 31 h), avoiding cell proliferation contribution to wound closure. ImageJ software (NIH, Bethesda, MD, USA) was used to measure the cell-free area, which was expressed as a percentage of wound closure calculated as (wound area at time 0 − wound area at time 20) × 100/wound area at time 0. Each experiment was performed at least four times, with two technical replicates per condition.

### 4.12. Determination of Intracellular Reactive Oxygen Species (ROS) Levels

ROS production was evaluated using the fluorescent probe 2′,7′-dichlorodihydrofluorescein diacetate (DCFH-DA), as previously described [7]. Upon cellular uptake, the non-fluorescent DCFH-DA is deacetylated by intracellular esterases to form 2′,7′-dichlorodihydrofluorescein (H_2_DCF), which is subsequently oxidized by ROS to generate the highly fluorescent compound 2′,7′-dichlorofluorescein (DCF).

For the assay, 7 × 10^3^ t-HESC cells and 12-Z cells per well were seeded in black 96-well optical-bottom microplates (Thermo Scientific #165305; Waltham, MA, USA). In one experimental setup, cells were pretreated with 0.1 μM hydrogen peroxide (H_2_O_2_) for 1 h and subsequently exposed to increasing concentrations of RE (8, 12, 15, 18, 25, and 30 μg/mL) or vehicle for an additional 24 h. After treatment, cells were incubated with 10 μM DCFH-DA for 30 min in the dark at 37 °C in a humidified atmosphere containing 5% CO_2_.

Fluorescence intensity, indicative of intracellular ROS levels, was measured using a microplate reader (excitation: 485 nm; emission: 530 nm). Changes in fluorescence (Δfluorescence) were calculated by subtracting the mean fluorescence intensity at 0 min from that measured at 70 min (t-HESC, *n* = 5; 12-Z, *n* = 6).

### 4.13. Statistical Analysis

Statistical analyses were performed using GraphPad Prism version 8.0 (GraphPad Software Inc., San Diego, CA, USA). Independent biological replicates were used for all statistical analyses. Normality and variance homogeneity were evaluated using the Shapiro–Wilk and Brown–Forsythe tests, respectively. Groups meeting both criteria were compared via one-way ANOVA with Tukey’s post hoc test. Otherwise, the nonparametric Kruskal–Wallis test with Dunn’s post hoc test was utilized. Data are expressed as mean ± SEM. Differences were considered statistically significant at *p* < 0.05.

## 5. Conclusions

In conclusion, this study underscores the value of a whole RE, whose biological activity may result from the concerted action of multiple phytochemicals across different cellular processes. Using immortalized in vitro models of endometriosis, we found that this diterpene-rich extract modulated key features of disease pathophysiology, including cell proliferation, cell death, cell cycle progression, and intracellular oxidative activity. While additional mechanistic studies and in vivo validation are required, these findings suggest the potential relevance of this phytocomplex for the development of future therapeutic or nutraceutical strategies for endometriosis management.

## Data Availability

The data supporting the findings of this study are available from the corresponding author upon reasonable request.

## Supplementary Materials

The following supporting information can be downloaded at https://www.mdpi.com/article/10.3390/ijms27135654/s1.

## Abbreviations

The following abbreviations are used in this manuscript: AAD, amino-actinomycin D; ABTS, 2,2-azino-bis-3-ethylbenzothiazoline-6-sulfonic acid; ANOVA, analysis of variance; ATCC, American type culture collection; CA, carnosic acid; CS, carnosol; DAD, diode array detector; DAPI, 4′,6-diamidino-2-phenylindole; DCF, 2′,7′-dichlorofluorescein; DCFH-DA, 2′,7′-dichlorodihydrofluorescein diacetate; DPPH, 2,2-diphenyl-1-picrylhydrazyl; DTT, dithiothreitol; EFSA, European Food Safety Authority; FBS, fetal bovine serum; FITC, Fluorescein isothiocyanate; FRAP, ferric reducing ability of plasma; GAPDH, Glyceraldehyde 3-phosphate dehydrogenase; H_2_DCF, 2′,7′-dichlorodihydrofluorescein; NIH, National Institutes of Health; PI, propidium iodide; RA, rosmarinic acid; RE, rosemary extract; ROS, reactive oxygen species; RP-HPLC, reversed-phase high-performance liquid chromatography; SDS-PAGE, Sodium dodecyl sulfate-polyacrylamide gel electrophoresis; TE, trolox equivalents; WST-1, Water-soluble tetrazolium salt-1.
